# Understanding the Processing Quality Problem for Cutting Ceramic Materials Using the Thermal-Controlled Fracture Method Induced by a Single-Surface Heat Source

**DOI:** 10.3390/mi15080957

**Published:** 2024-07-26

**Authors:** Xiaoliang Cheng, Zhenzhen Cui, Junwen Chen, Yang Wang, Lijun Yang

**Affiliations:** 1 School of Mechanical Engineering, Hubei University of Automotive Technology, Shiyan 442002, China; chenjw@huat.edu.cn; 2 School of Automobile and Transportation, Wuxi Institute of Technology, Wuxi 210044, China; 3 School of Mechatronics Engineering, Harbin Institute of Technology, Harbin 150001, China; wyyh@hit.edu.cn (Y.W.); yljtj@126.com (L.Y.)

**Keywords:** ceramic materials, thermal-controlled fracture method, surface heating mode, processing problem, trajectory deviation, simulation, uneven distribution, fracture quality

## Abstract

The thermal-controlled fracture method has been increasingly focused upon in the high-quality cutting of advanced ceramic materials due to its excellent characteristics. The successful application of this method in cutting ceramics mainly depends on the volumetric heating effect. However, most ceramics are treated using the surface heating mode. For the surface heating mode, the processing quality, including fracture trajectory and fracture quality, is far lower than the industrial application standards. This work was conducted to reveal the mechanism of this processing quality. Experiments involving cutting ceramics in single-surface heating mode indicate that the fracture trajectories of the upper and lower surfaces display a significant inconsistency, and the fracture quality is worse than that using the dual-surface heating mode. A cutting model was established to calculate the thermal stress distribution and to simulate the crack-propagation behaviors. The simulation results show good agreement with the experiment and provide the stress distribution, and are used to understand the reason for the processing quality problem. The mechanism of the trajectory deviation and uneven distribution of the fracture quality is revealed based on the simulation and calculation results. This study helps provide a deep understanding of the processing problems arising from this method and thus helps to innovate high-quality processing methods in this field.

## 1. Introduction

Ceramics have been increasingly used in frontier domains such as aerospace and advanced chips due to their properties of high hardness, low thermal expansion, and excellent stability [1]. High-quality cutting technology is the foundation for preparing qualified parts from ceramic materials. However, the high hardness of ceramic materials can cause severe wear of tools in contact force cutting mode, as well as serious damage to the surface and subsurface of the machined workpiece [2,3,4,5]. Laser cutting methods can also cause the formation of heat-affected zones in the kerf, which can weaken the workpiece [6,7]. This severe damage induced by conventional cutting methods at the fracture surface requires complex subsequent processing to achieve qualified parts, causing a significant increase in manufacturing costs, which hinders the widespread application of advanced ceramic materials in industrial fields [8,9,10].

In 1968, the thermal-controlled fracture method (TCFM) was proposed by Lumley, which utilizes thermal stress to guide the crack propagation on brittle materials to cut materials. TCFM can achieve high cutting quality at relatively low temperature (no more than 350 °C) without damage in the kerf and without material removal [11]. After decades of development, this method has been implemented in industrial production for cutting flat panel display glass [12]. The successful application of TCFM in cutting glass depends on the volumetric heating effect caused by a specific wavelength laser irradiating glass [13]. However, the research on cutting opaque ceramic materials, such as laser cutting Al_2_O_3_ ceramics and microwave cutting low-dielectric ceramics, indicated that the surface heating mode causes poor processing quality [14,15]. The processing quality problems in cutting ceramic materials using TCFM under surface heating conditions mainly include whether the fracture trajectory follows the ideal direction and whether the fracture quality is good enough and uniform.

Regarding fracture trajectory, Brugan used a dual-beam CO_2_ continuous laser to cut Al_2_O_3_ ceramics with a thickness of 2.54 mm using TCFM. The results indicated that the TCFM could achieve the cutting of Al_2_O_3_ ceramics in surface heating mode, and induced problems of trajectory deviation [16]. lu used microwaves to cut ceramic materials coated with graphite using TCFM, and the mechanism of uncontrolled crack propagation was studied [17]. Cheng studied the crack-propagation behavior in cutting silicon wafers using TCFM via a surface heat source induced by a laser, and proposed a method of surface pre-cracks to guide crack propagation to achieve approximate linear crack propagation [18].

Regarding fracture surface quality, Ueda used TCFM to cut crystalline silicon, Al_2_O_3_ ceramics, Si_3_N_4_ ceramics, etc. The results showed that the surface roughness of the fracture surface could reach 0.7 μm, 1.3 μm, and 100 μm, respectively [19]. Cai used TCFM to cut glass/silicon/glass sandwich materials, and the surface roughness of the fracture surface of the silicon layer under the action of a surface heat source reached 1 μm [20]. Saman used TCFM to cut different kinds of ceramics and studied the stress distribution characteristics. The results indicated that if the material absorbs a laser on a single surface, the maximum tensile stress area can easily be located on the back of the material, which seriously affects the stable propagation of cracks. Saman inferred that it is difficult to obtain good processing quality using the surface heating mode due to these stress distribution characteristics [21].

The above research indicates that cutting ceramics by TCFM can achieve ideal processing quality. However, the processing quality under the surface heating mode is far lower than that in volumetric heating mode, and should be improved for industry applications. To the authors’ knowledge, there has been little research to date on the mechanism inducing poor processing quality using TCFM under a surface heat source. The crack-propagation mode and material fracture mechanism under the influence of a surface heat source have not been revealed. This hinders the improvement and optimization of the cutting quality in this mode.

In this study, microwaves were used to cut ceramic materials using TCFM under a surface heat source. The influence of processing parameters on fracture trajectory and fracture surface quality was studied. A TCFM cutting model was established to simulate the fracture behaviors. Combining experimental and simulation results, the mechanism of trajectory deviation and uneven distribution of fracture quality in cutting ceramic materials using TCFM under a surface heat source was revealed. This work helps to understand the mechanism of trajectory deviation and poor fracture surface quality in the cutting of ceramic materials using TCFM, and thus helps to put forward methods for improving the cutting quality under a surface heat source.

## 2. Experiment

### 2.1. Experimental Method

**Figure 1:**
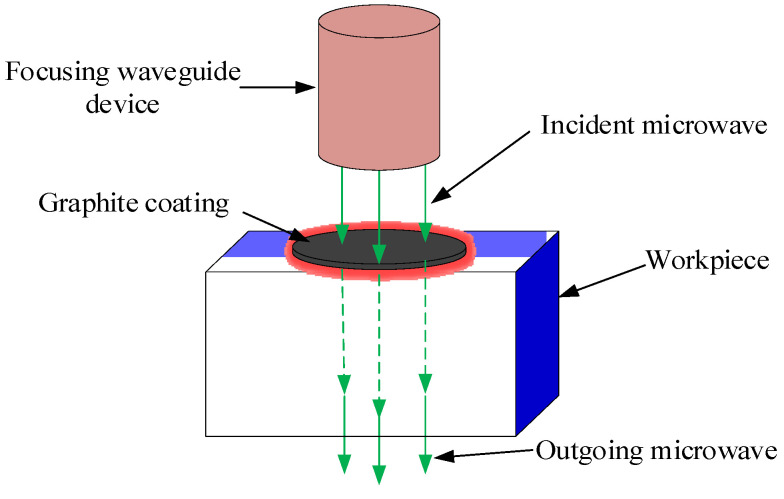
Schematic diagram of surface heating mode induced by microwaves.

Al_2_O_3_ ceramic is opaque and does not absorb a laser, thus making it difficult to apply a volumetric heat mode. Because of its low dielectric property, it is also difficult for microwaves to interact with this ceramic. In this work, graphite with high permittivity was used to coat one surface of the Al_2_O_3_ ceramic to absorb microwaves to generate a surface heat source. Figure 1 shows the principle of producing the surface heating mode using the coating material induced by microwaves. The unabsorbed microwaves pass the ceramic body and enter the environment through the lower surface. The uneven heat distribution generated by the high electromagnetic loss on the coating material produces a thermal stress field. The cutting of the ceramic is realized by moving the thermal stress to guide crack propagation.

A pre-crack was carved on the workpiece using a diamond wire with a diameter of 0.25 mm, which produced a stress amplification effect. The pre-crack with a depth of 0.1 mm is the start point of crack propagation. When the tensile stress exceeds the fracture limit at the pre-crack, the crack system reaches the propagation condition. When the waveguide is given an appropriate moving speed relative to the workpiece, the crack propagates forward and realizes cutting.

### 2.2. Experimental Material and Apparatus

**Figure 2:**
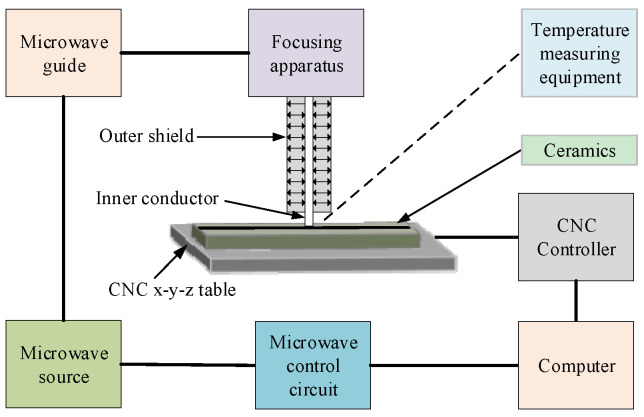
Schematic of microwave cutting machine using the thermal-controlled fracture method.

The experiment was conducted on a microwave cutting machine. Figure 2 shows the schematic of the microwave cutting machine in TCFM. The microwave with a specific frequency is produced from the microwave source. Then, it is modulated by the microwave guide into a focusing apparatus. Finally, the focusing apparatus outputs the microwave, which is suitable for heating the material from the inner conductor. The inner conductor is surrounded by the outer shield to focus the microwave to achieve higher energy density. The coating surface of the workpiece is just below the inner conductor. The NC motion device with the x-y-z direction is integrated into the cutting machine to realize cutting movement. The z-axis is used to adjust the distance between the inner conductor and the workpiece. The x-axis is used to realize cutting movement, and the y-axis is used to center the heat source and the workpiece. This cutting device is produced by Nanjing Huiyan Microwave Equipment Company in China. It is used to cut brittle material and is equipped with safety facilities such as a filter screen to ensure safety. The microwave frequency of this device is 2.45 GHz and the maximum output power is 1500 W.

**Figure 3:**
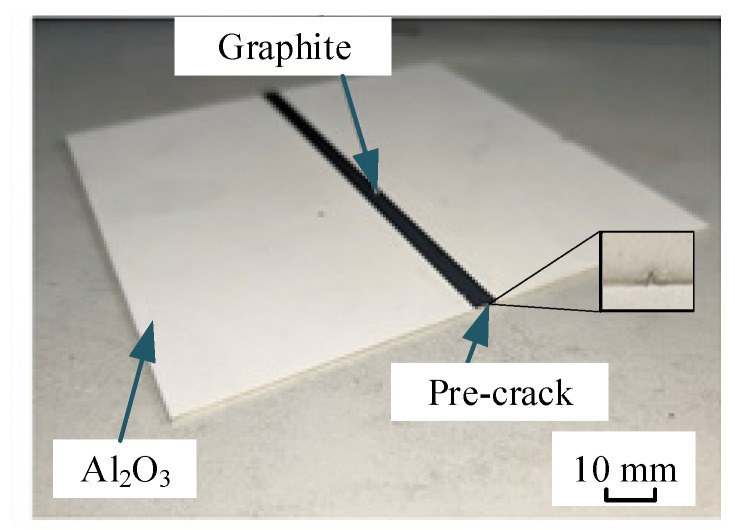
Al_2_O_3_ ceramic plate coated with graphite.

Figure 3 shows the Al_2_O_3_ ceramic plate used in this study. Graphite powder with a thickness of 0.1 mm and a width of 1 mm is coated on the expected cutting path. The plate is 100 mm × 100 mm in size. The graphite powder with a micron-size (mesh of 8000 and particle size of 1.6 μm) is mixed with alcohol. The mixture is sprayed evenly by an electric sprayer whose power comes from an air pump. As shown, a pre-crack is made on the end of the plate.

The microwave power and scanning speed were used in the experiments as processing parameters. Table 1 shows the variation range of these processing parameters. The crack propagation of the initial segment was observed by a Stemi 305 optical microscope, which was produced by CARI ZEISS in Oberkochen of Germany and can magnify objects clearly by more than 120 times.

**Figure 4:**
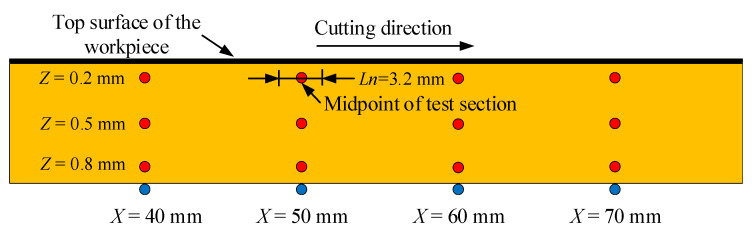
Position of the surface roughness test.

To investigate the distribution characteristics of the fracture surface quality, some measurement sites were set on the section. Figure 4 shows the location of the measurement sites in the experiments. A plane on the surface heat source in workpiece was selected as the reference. Three depth positions, which are 0.2 mm, 0.5 mm, and 0.8 mm from the reference plane in the direction of the thickness, were selected to measure the surface roughness. To achieve stable fracture quality and avoid the influence of unstable boundary conditions, four horizontal inspection positions, namely 40 mm, 50 mm, 60 mm, and 70 mm from the inlet along the cutting direction, were selected. To ensure the value of the experimental results, each experiment was repeated four times, and the average of the results was taken.

## 3. Results

### 3.1. Fracture Trajectory

**Figure 5:**
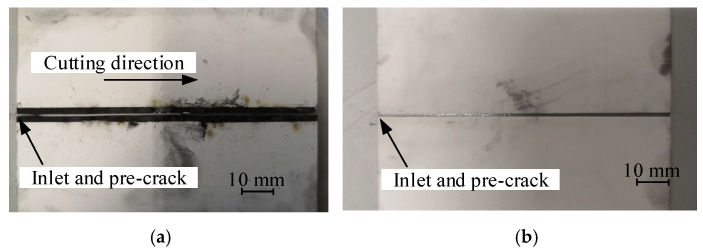
Experimental results of fracture trajectories of surface-heat-source cutting Al_2_O_3_ ceramic under using thermal-controlled crack propagation induced by microwaves: (**a**) the upper surface; (**b**) the lower surface.

Figure 5 shows the fracture trajectories at each surface of the Al_2_O_3_ ceramic plates after cutting at a microwave power of 1000 W and a scanning speed of 3 mm/s. It can be shown that the fracture trajectory approaches a straight line when cutting ceramics with a surface heat source. Comparing the processing effect of graphite in this study and clay as the microwave-absorbing material in the literature [22], it is notable that pure graphite micron powder can achieve better processing quality.

**Figure 6:**
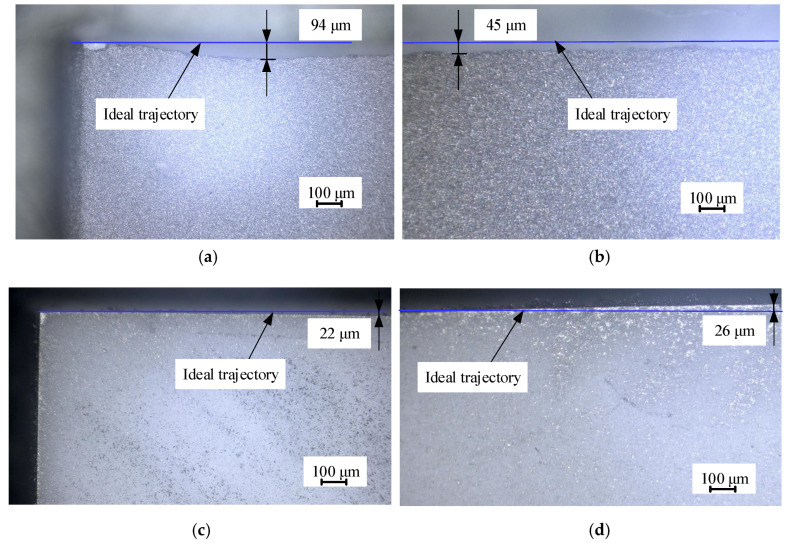
Optical micrograph of deviation in initial crack propagation: (**a**) inlet of upper surface; (**b**) extension of upper surface; (**c**) outlet of lower surface; (**d**) initial extension of lower surface.

Figure 6 shows a photo magnified with an optical microscope of the fracture trajectory on the two surfaces of the workpiece. It can be shown that the fracture trajectories of the upper and lower surfaces display significant inconsistencies from a microscopic perspective.

**Figure 7:**
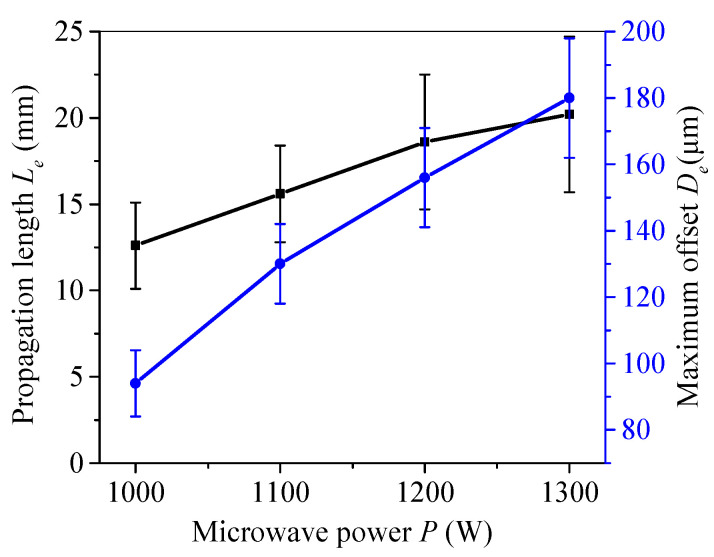
Effect of microwave power on the offset propagation length and the maximum offset extension.

The effect of processing parameters on fracture trajectory was studied. Figure 7 shows the relationship between the microwave power and the trajectory deviation of the initial segment. It shows the effect of the microwave power on the initial trajectory deviation length *Le* and maximum offset *De* of the upper and lower surfaces at a cutting speed of 3 mm/s and a microwave power of 1000 W, 1100 W, 1200 W, and 1300 W. As is shown, the deviation length *Le* and maximum offset *De* both increase as the microwave power increases, and the *De* increases more significantly. Therefore, it is necessary to choose an appropriate microwave power in order to reduce the trajectory deviation.

### 3.2. Fracture Surface Quality

**Figure 8:**
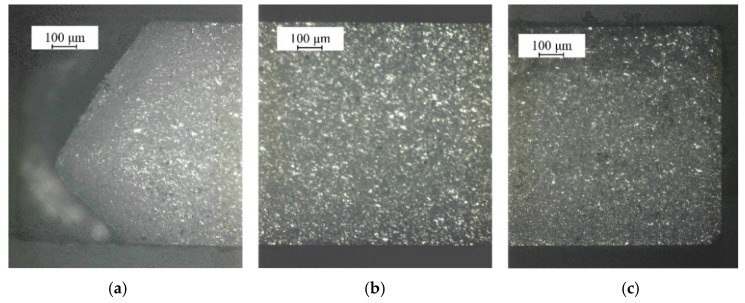
Experimental results of fracture surface of Al_2_O_3_ ceramics under the action of thermally induced crack propagation using a surface heat source:(**a**) the cutting inlet; (**b**) the middle segment; (**c**) the cutting outlet.

Figure 8 show the fracture surface of cutting Al_2_O_3_ ceramic plates at a microwave power of 1000 W and a scanning speed of 3 mm/s. As is shown, there is obvious processing damage at the entrance due to the process of prefabricating cracks using a diamond wire saw. It is notable that the fracture quality of the middle and outlet sections is better than that at the entrance, and the middle section is the best. There is a small amount of edge damage at the outlet.

**Figure 9:**
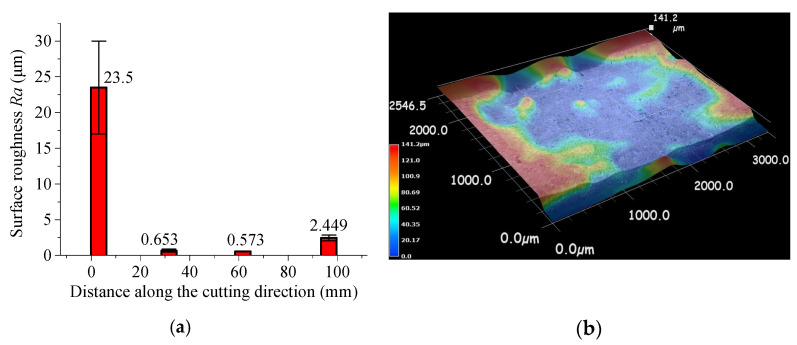
Surface roughness and fracture surface along the scanning direction at the middle depth: (**a**) surface roughness; (**b**) 3D figure of fracture surface.

Figure 9 shows the variation in the arithmetic mean deviation of surface roughness *Ra* at the middle thickness of the workpiece along the cutting direction, ranging from 1.5 mm to 4.7 mm, 30 mm to 33.2 mm, 60 mm to 63.2 mm, and 95 mm to 98.2 mm, when the microwave power is 1000 W and the cutting speed is 3 mm/s. The sampling length is 0.8 mm, and the evaluation length is 3.2 mm. It is notable that the surface roughness *Ra* of the initial scanning segment is 1 to 2 orders of magnitude higher than that of the middle and end segments, which corresponds to the inconsistent propagation of surface cracks on the upper and lower surfaces.

**Figure 10:**
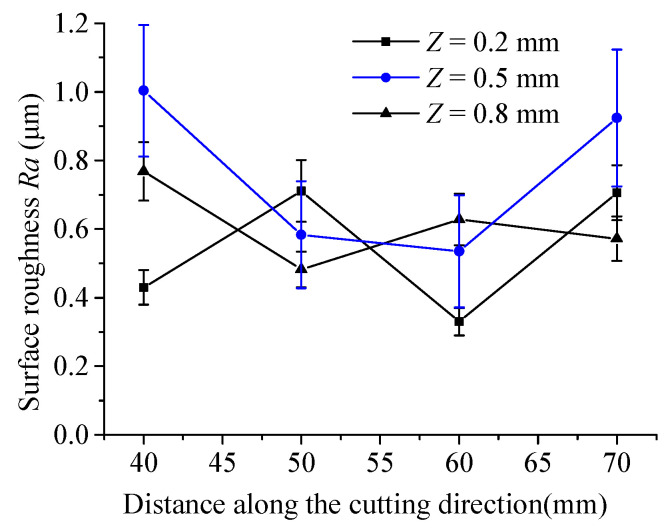
Surface roughness distribution at different positions of the material at the middle segment.

Figure 10 shows the variation in surface roughness *Ra* in the thickness direction of the workpiece. As is shown, the average *Ra* at most points is between 0.2 μm to 1 μm. It is notable that there is a significant difference in *Ra* along the depth direction at various cutting positions without obvious regularity.

**Figure 11:**
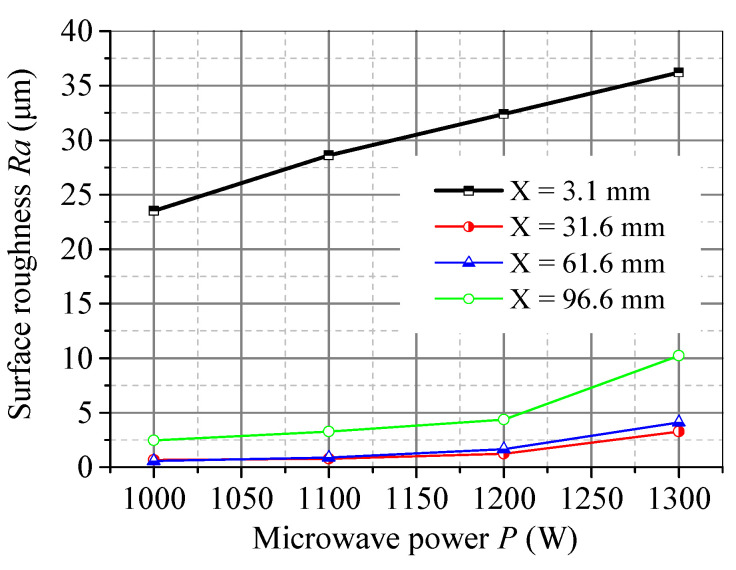
Effect of microwave power on the surface roughness along the scanning direction at different positions in the middle depth of the material.

Figure 11 shows the effect of microwave power on *Ra* under the condition of a cutting speed of 3 mm/s at several positions along the cutting direction. It indicates that the surface roughness of each position increases as the microwave power increases. The microwave power has a significant impact on the surface roughness of the initial segment. When the maximum microwave power is 1300 W, the surface roughness of both the initial and final segments will increase significantly. Therefore, it is necessary to choose an appropriate microwave power when using a surface heat source for cutting.

## 4. Discussion

### 4.1. Finite Element Modeling

The main physical processes of cutting ceramic materials using TCFM are interactions between energy beams and matter, heat generation, heat transfer, thermal stress generation, and crack propagation.

Since cracks tend to propagate perpendicularly to the maximum transverse tensile stress component, which is affected by the stress intensity factor in different fracture modes, the fracture problem is transformed into determining the maximum value of the mechanical energy release rate in fracture mechanics.

The crack-propagation condition is that the tensile stress at the tip of the pre-crack exceeds the fracture limits of the material. From an energy perspective, it is a process of converting elastic energy into surface energy of the fracture surface.

The cutting of ceramics materials by TCFM is a complex physical process. It is difficult to calculate the temperature, thermal stress, and crack propagation using an analytical model. A finite element modeling (FEM) technology with the aid of ABAQUS 6.14 software was used in this study to calculate these physical quantities and simulate the cutting process.

**Figure 12:**
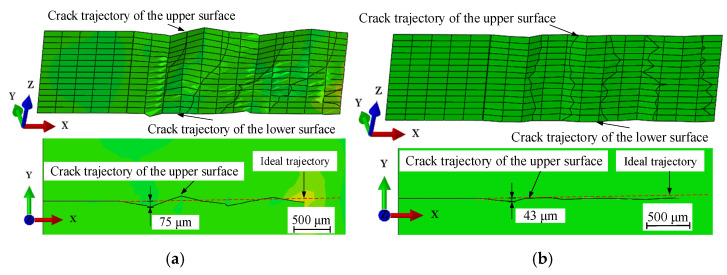
Simulation of initial crack-propagation path and section quality via the thermal cracking method using a surface heat source and its comparison with a volumetric heat source mode: (**a**) crack propagation induced by a surface heat source using the thermal cracking method; (**b**) crack propagation induced by a volumetric heat source using the thermal cracking method.

The cutting simulation was conducted for both surface and volumetric heat sources. Figure 12 shows the simulation results of crack propagation for cutting Al_2_O_3_ ceramics by a microwave surface heat source and the crack propagation for cutting glass with a volumetric heat source. From Figure 12a, it is notable that the crack propagates inconsistently between the upper and the lower surface of the workpiece. The crack propagation on the upper surface presents a significant folding line, while the lower surface is relatively straight. From Figure 12b, it is notable that that the crack-propagation paths on the upper and lower surfaces of the workpiece are consistent under the action of a volumetric heat source, and the crack-propagation paths tend to be straight.

**Figure 13:**
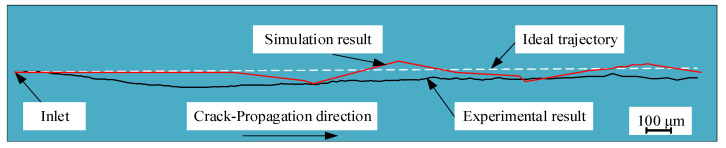
Comparison of experimental and simulation results of the initial crack-propagation trajectory.

Figure 13 show a comparison between the experimental and simulation results of the crack-propagation trajectory under a surface heat source. It shows that the degree of offset generated in experiments and simulations is relatively close. Therefore, the simulation of crack propagation in this study is suitable for discussing the cutting quality.

### 4.2. Trajectory Deviation Mechanism

**Figure 14:**
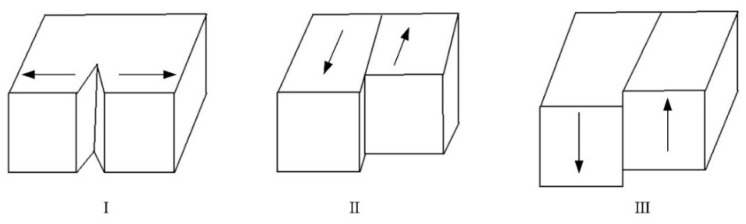
Three basic fracture modes.

There are three basic modes of crack propagation and fracture, which are type I (open), type II (sliding), and type III (tear). Figure 14 shows a schematic of these three basic fracture modes. Among them, type I means that the crack only breaks under the action of tensile stress perpendicular to the fracture surface; type II means that the crack breaks only under the action of longitudinal shear stress perpendicular to the leading edge of the crack; type III means that the crack breaks only under the action of lateral shear stress parallel to the leading edge of the crack.

Among the three basic fracture modes, type I is closest to the fracture of materials with high brittleness. Due to the high brittleness of the ceramic materials in this study, the fracture mode is generally considered to be type I. However, during the fracture and propagation of the actual crack, it will be affected by both the sliding and tear of the others. In this way, the crack is usually a “tilted” (type I and type II composite) fracture or “torsional” (type I and type III composite) fracture according to the actual influence of these two effects during the crack-propagation process.

**Figure 15:**
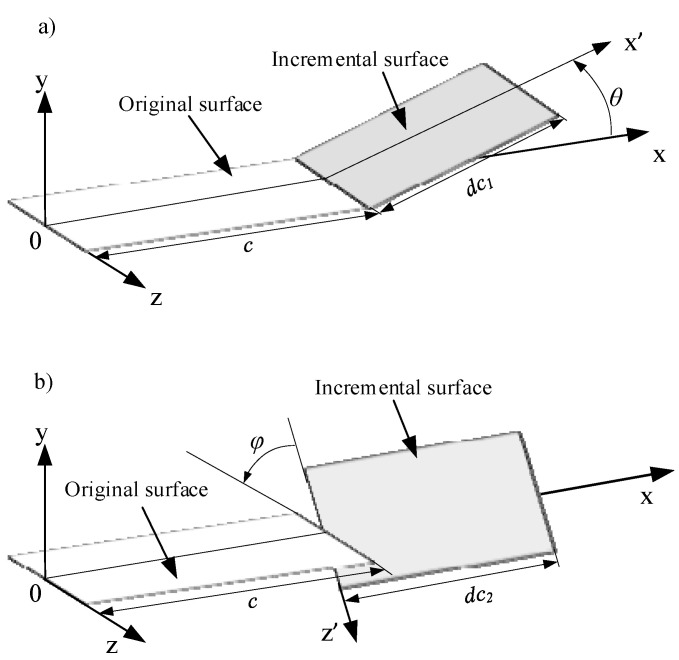
Two typical deviation types of crack propagation: (**a**) inclined type; (**b**) torsion type.

**Figure 16:**
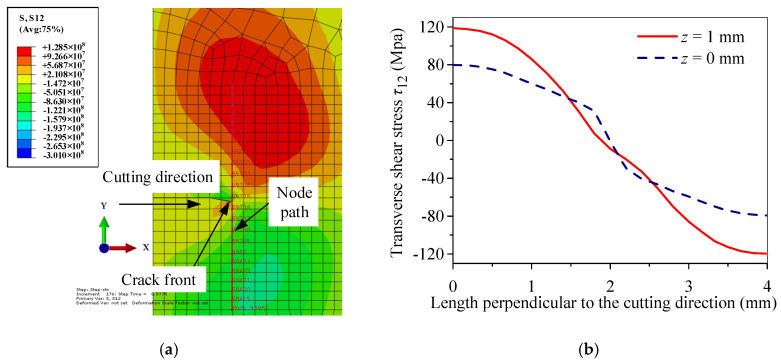
Transverse shear stress distribution front perpendicular to the scanning direction of a surface heat source in a Al_2_O_3_ ceramic surface crack at *t* = 1.9058 s: (**a**) cloud chart of transverse shear stress distribution and node path on the upper surface of the workpiece; (**b**) transverse shear stress distribution of the crack front perpendicular to the scanning direction.

The main reasons for the occurrence of offset propagation of cracks are divided into “inclined” type (type I and type II composite) fractures or “torsional” type (type I and type III composite) fractures. Figure 15 shows the schematic diagrams of these two typical types. It adopts the same coordinate system and crack location as the simulation calculation, where the “inclined” type in Figure 16a refers to the original crack surface producing a deflection angle *θ* relative to the original propagation direction under the influence of transverse shear force, and an extended length *dc_1_* of the included angle. The “torsional” propagation in Figure 15b is caused by the influence of lateral shear force on the original crack surface, resulting in a deflection angle *φ* relative to the original propagation direction, and the extended length *dc*_2_ of the included angle.

In order to study the mechanism of deviation of crack propagation in a surface heat source-induced thermal fracture, it is necessary to analyze the two kinds of shear stress near the crack that cause the migration. These are *S*_12_ and *S*_13_ in the output of the simulation results, which present the shear stress $\tau_{12}$ causing “inclined” propagation and shear stress $\tau_{13}$ causing “torsional” propagation.

Figure 16 shows the distribution of transverse shear stress $\tau_{12}$, which is perpendicular to the cutting direction at the crack front at *t* = 1.9058 s. To obtain effective data, a grid segment with a length of 2 mm was symmetrically taken on the front edge of the crack perpendicular to the initial crack plane, and the center position of this segment is the ideal position for the crack propagation without deviation. The number of selected nodes is 23, and the path direction of the selected nodes is in the positive y-axis direction. As is shown in Figure 16a, the crack on the upper surface of the workpiece shifted to the right. Figure 16b shows that $\tau_{12}$ of the upper surface changes more significantly than the lower surface, which is caused by the surface heat source being located on the upper surface of the workpiece. It is notable that the intersection of the $\tau_{12}$ curve and the 0 MPa line is close to the right side of the symmetry line. According to the theory of fracture mechanics, the crack always tends to propagate where the shear stress is the smallest, so the upper surface crack has a deviation to the right.

**Figure 17:**
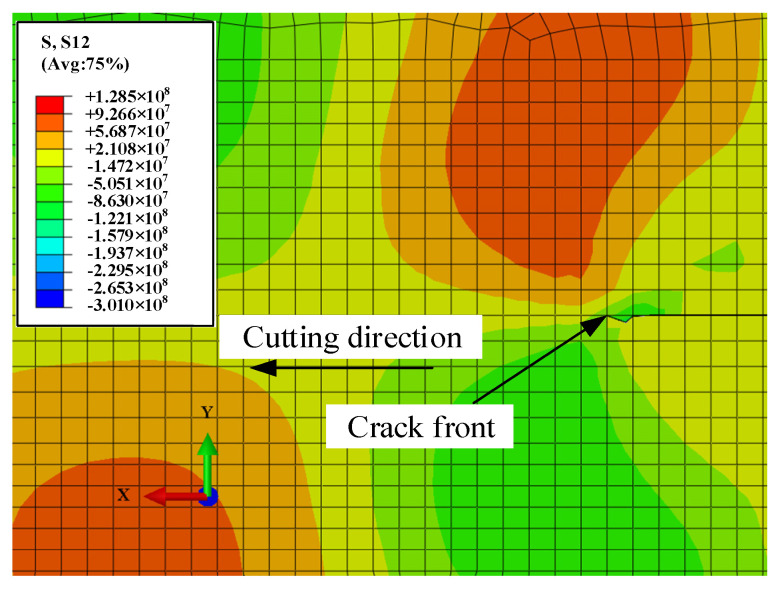
Crack propagation on the lower surface of the workpiece at *t* = 1.9058 s.

**Figure 18:**
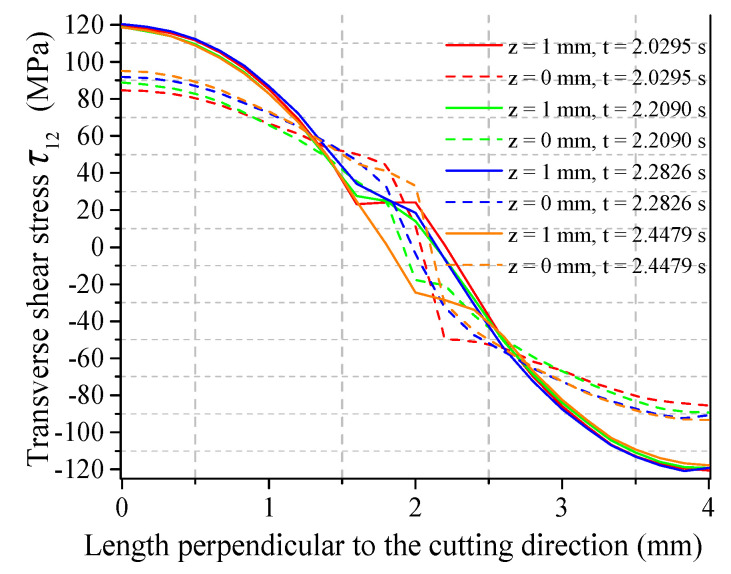
Transverse shear stress distribution at the upper and lower surfaces of the crack front at different times.

Figure 17 shows the crack propagation on the lower surface of the workpiece. It indicates that the crack on the lower surface of the workpiece is returning to the ideal propagation path. This is consistent with the middle position of the intersection of the $\tau_{12}$ curve and the 0 MPa line. Figure 18 shows the $\tau_{12}$ curve on each surface of the workpiece at multiple times during the subsequent crack-propagation process. It is notable that the significant size differences of $\tau_{12}$ on the upper and lower surfaces of the workpiece result in their different effects on the tilting behavior of the cracks. This is the reason for the inconsistent crack propagation on upper and lower surfaces, as well as their different deviation behaviors in experiments.

**Figure 19:**
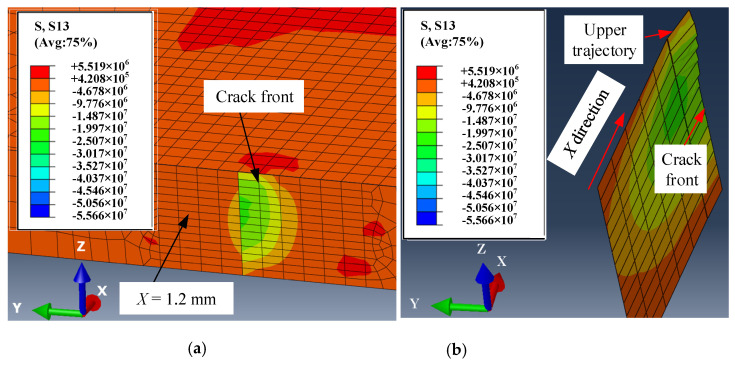
Lateral shear stress distribution near the front edge of the crack when the crack propagates with torsion at *t* = 1.7675 s: (**a**) distribution of lateral shear stress near the crack front; (**b**) torsional propagation of the crack.

Figure 19 shows the lateral shear stress distribution near the crack front edge at *t* = 1.7675 s. The plane X = 1.2 mm perpendicular to the x-axis is made at the crack front edge to display the lateral shear stress distribution shown in Figure 19a. It is notable that the lateral shear stress has a significantly asymmetric distribution within a range of 400 μm around the front edge of the crack. The influence of this lateral shear stress distribution on the formation near the crack front leads to torsional propagation, as shown in Figure 19b. Due to the direct heating effect on the upper surface of the workpiece, the twisting behavior near the upper surface is more pronounced. The trajectory deviation occurs under the combined action of lateral shear stress and transverse shear stress in cutting ceramics with a surface heat source.

### 4.3. Uneven Mechanism of Fracture Surface Quality

**Figure 20:**
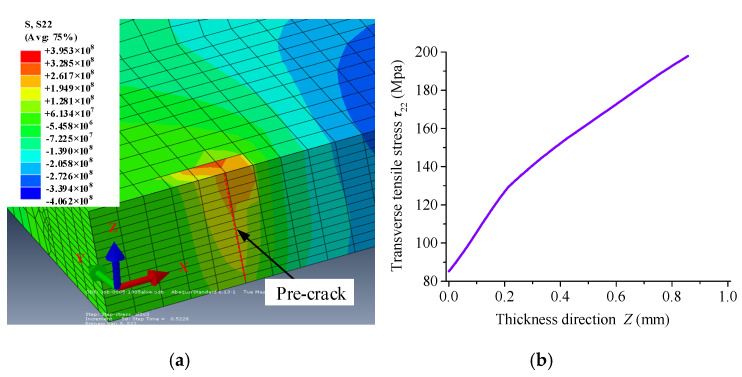
Distribution of transverse tensile stress at the crack front at the moment of propagation: (**a**) circumferential condition of crack front edge; (**b**) crack front edge.

The fracture surface is formed under the guidance of transverse tensile stress whose characteristic determines the mode of crack propagation in TCFM. Figure 20 shows the distribution of transverse tensile stress at the crack front and its circumferential condition when the crack is about to propagate.

It indicates that the stress gradient near the crack front is relatively large when it is about to propagate, and the transverse tensile stress is large near the surface under the condition of a surface heat source.

The gradient of transverse tensile stress along the thickness direction of the material indicates that crack propagation is severely asynchronized at different positions of the cross-section. As was reported in [18], the propagation first occurs from the root of the existing crack on the upper surface, and the crack expands to the lower surface along the thickness direction. The incentive for its subsequent expansion is that the expanding crack forms a new tip with the existing crack in the vertical direction, which has the minimum curvature radius and the most concentrated stress to guide the crack to propagate downwards. This crack-propagation mode results in the uneven distribution of the fracture quality in the cutting experiment.

**Figure 21:**
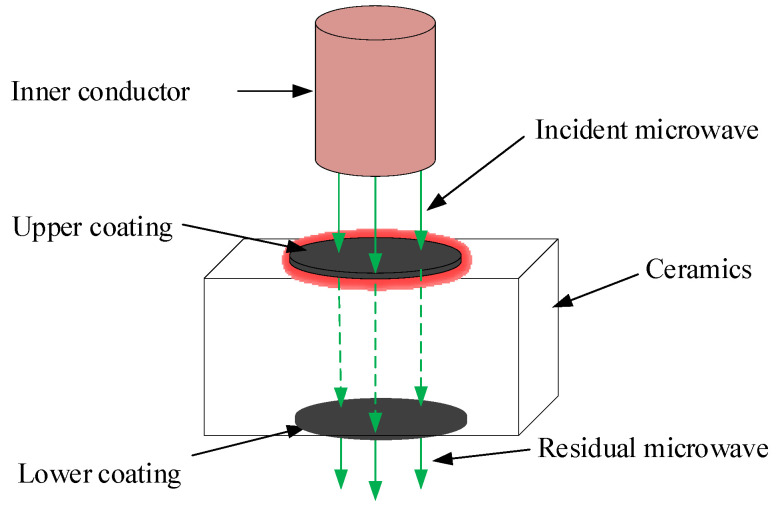
Schematic diagram of heating ceramics using a microwave dual-surface heat source.

To improve the large stress gradient in single-sided surface heating mode, a dual-sided surface heating method was proposed by the authors of this article in the literature [23]. Figure 21 shows the schematic diagram of heating ceramics using a microwave dual-surface heat source. The lower surface of the workpiece is coated by graphite, which is used to absorb the underutilized microwaves and forms a lower surface heat source.

**Figure 22:**
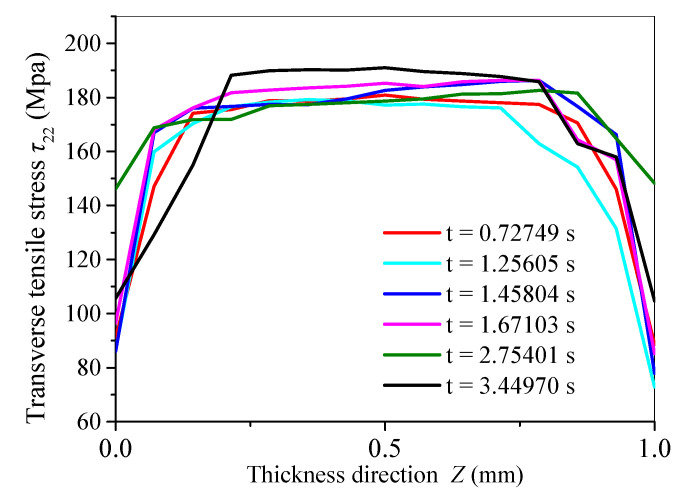
Distribution of transverse tensile stress along the thickness direction at the crack front at the moment of propagation under the action of a dual-surface heat source at different times.

Figure 22 shows the transverse tensile stress at the crack front along the direction of material thickness at the moment of propagation under the action of a dual-surface heat source at different times. As is shown, the maximum transverse tensile stress zone under the action of a dual-surface heat source is located at the middle section of the crack root. This indicates that the difference in the maximum transverse tensile stress zone and its gradient at the crack front edge under different heat sources determines their different crack-propagation modes and their fracture quality. According to this study, the distribution of the transverse tensile stress at the crack front edge determines the distribution of fracture quality.

**Figure 23:**
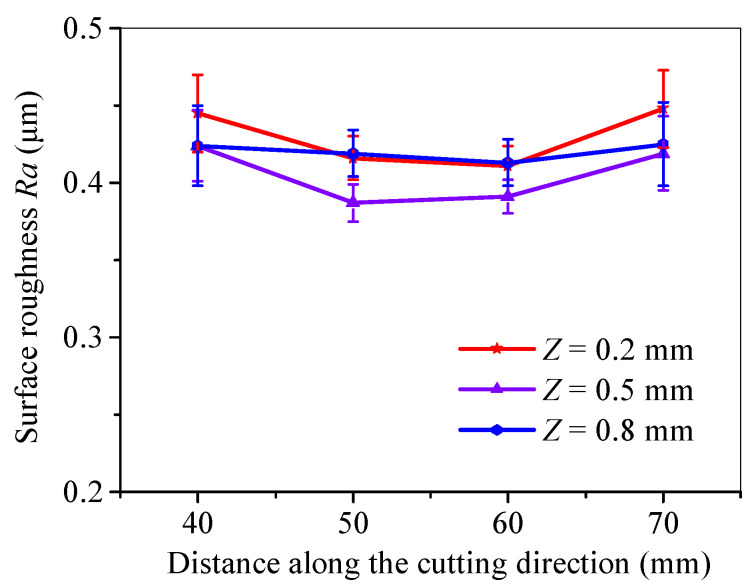
Surface roughness distribution at the middle segment under dual-surface heat mode.

Cutting experiments under a dual-sided surface heat source were conducted. Figure 23 shows the surface roughness distribution at the middle segment at the condition of 600 W of microwave power and a cutting speed of 3 mm/s under dual-surface heat mode. As is shown, the fracture quality and its distribution under the action of a dual-sided surface are significantly better, and the distribution is more uniform than that of a single-surface heat source. The middle depth of the section has the best fracture quality, which has good agreement with that revealed in reference [19].

## 5. Conclusions

In this work, experiments and simulations of cutting ceramics using the thermal-controlled fracture method with a surface heat source were conducted to reveal the mechanism of trajectory deviation and uneven distribution of fracture quality. Some conclusions can be made from this study, as follows.

A finite element model was established to calculate the physical quantities and simulate the cutting process in the thermal-controlled fracture method in surface heating mode. The crack-propagation results of the simulation were consistent with the trend of the cutting path in the experiment. The modeling results could be used to reveal the mechanism of trajectory deviation and uneven distribution of fracture quality for this method.

The trajectory deviation occurs under the combined action of lateral shear stress and transverse shear stress in cutting ceramics with a surface heat source.

The significant differences in $\tau_{12}$ on the upper and lower surfaces of the workpiece result in their different effects on the tilting behavior of the cracks and the inconsistent crack propagation. This is the reason for the trajectory deviation under a surface heat source.

The significantly asymmetric distribution of lateral shear stress around the front edge of the crack leads to torsional propagation under a surface heat source.

The distribution of the transverse tensile stress along the thickness direction of the workpiece shows a positive correlation with the uneven distribution of fracture quality in the single-surface heating mode, in contrast with the dual-sided surface and volumetric heat source cutting method.

Future research could focus on innovative techniques that could reduce the gradient of transverse tensile stress in the thickness direction and correct the asymmetric distribution of the two types of shear stress.

## Figures and Tables

**Table 1:** Variation range for processing parameters using TCFM by a surface heat source induced by microwaves.

Test Group No	Microwave Power (W)	Scanning Speed (mm/s)
NO.1	600–1200	2.0–3.5
NO.2	900–1500	0.5–2.0
NO.3	1200–1500	0.3–0.6

## Data Availability

Data available on request from the authors.

## References

[1] Sun J., Yu H., Zeng D., Shen P. (2022). Wire–powder–arc additive manufacturing: A viable strategy to fabricate carbide ceramic/aluminum alloy multi-material structures. Addit. Manuf..

[2] Rakshit R., Das A. (2019). A review on cutting of industrial ceramic materials. Precis. Eng..

[3] Lin H., Zhou M., Wang H., Bai S. (2023). Investigation of Cutting Force and the Material Removal Mechanism in the Ultrasonic Vibration-Assisted Scratching of 2D-SiCf/SiC Composites. Micromachines.

[4] Wu Q., Zhou X., Pan X. (2023). Cutting tool wear monitoring in milling processes by integrating deep residual convolution network and gated recurrent unit with an attention mechanism. Proc. Inst. Mech. Eng. Part B.

[5] Chen M., Zhang S., Wu Y., Wang H. (2023). Build an accurate 3D geometrical model of a soft knife profile of abrasive water jet. The Int. J. Adv. Manuf. Tech..

[6] Yang L.J., Ding Y., Cheng B., Mohammed A., Wang Y. (2017). Numerical simulation and experimental research on reduction of taper and HAZ during laser drilling using moving focal point. Int. J. Mach. Tool. Manu..

[7] Li C., Hu Y., Zhang F., Geng Y., Meng B. (2023). Molecular dynamics simulation of laser assisted grinding of GaN crystals. Int. J. Mech. Sci..

[8] Li C., Piao Y., Zhang F., Zhang Y., Hu Y., Wang Y. (2023). Understand anisotropy dependence of damage evolution and material removal during nanoscratch of MgF_2_ single crystals. Int. J. Extreme Manuf..

[9] Li C., Piao Y., Meng B., Hu Y., Li L., Zhang F. (2022). Phase transition and plastic deformation mechanisms induced by self-rotating grinding of GaN single crystals. Int. J. Mach. Tool. Manuf..

[10] Lin W., Yu D., Zhang C., Zhang S., Tian Y., Liu S., Luo M. (2017). Multi-objective optimization of machining parameters in multi-pass turning operations for low-carbon manufacturing. Proc. Inst. Mech. Eng. Part B.

[11] Lumley R.M. (1968). Controlled Separation of Brittle Materials Using a Laser. Am. Ceram. Soc. Bull..

[12] Kim K.R., Kim J.H., Farson D.F., Choi H.W., Kim K.H. (2008). Hybrid laser cutting for flat panel display glass. Jpn. J. Appl. Phys..

[13] Zhao C., Zhang H., Wang Y. (2014). Semiconductor laser asymmetry cutting glass with laser induced thermal-crack propagation. Opt. Laser. Eng..

[14] Tsai C., Chen H. (2003). Laser cutting of thick ceramic substrates by controlled fracture technique. J. Mater. Process. Technol..

[15] Wang H., Zhang H., Wang Y. (2016). Splitting of glass and SiC ceramic sheets using controlled fracture technique with elliptic microwave spot. Ceram. Int..

[16] Brugan P., Cai G., Akarapu R., Segall A.E. (2006). Controlled-fracture of prescored alumina ceramics using simultaneous CO_2_ lasers. J. Laser. Appl..

[17] Lu Y., He Z., Xu J., Wang Y., Yang L. (2023). A novel method by microwave cutting ceramics based on thermal crack and trajectory control. Int. J. Adv. Manuf. Tech..

[18] Cheng X., Yang L., Wang M., Cai Y., Wang Y., Ren Z. (2019). Laser beam induced thermal-crack propagation for asymmetric linear cutting of silicon wafer. Opt. Laser. Technol..

[19] Ueda T., Yamada K., Oiso K., Hosokawa A. (2002). Thermal Stress Cleaving of Brittle Materials by Laser Beam. Cirp. Ann. Manuf. Technol..

[20] Cai Y., Wang M., Zhang H., Yang L., Fu X., Wang Y. (2017). Laser cutting sandwich structure glass–silicon–glass wafer with laser induced thermal—Crack propagation. Opt. Laser. Technol..

[21] Saman A.M., Furumoto T. (2018). Evaluation of Separating Process for Different Materials by Thermal Stress Cleaving Technique. Int. J. Precis. Eng..

[22] Wang H., Zhang H., Wang Y., Wang M. (2019). Thermal controlled fracture of Al_2_O_3_ substrate by inducing microwave discharge in graphite coat. Ceram Int..

[23] Cheng X., He Z., Wang H., Wang Y. (2022). Splitting Opaque, Brittle Materials with Dual-Sided Thermal Stress Using Thermal-Controlled Fracture Method by Microwave. Crystals.

